# Maternal Caffeine Intake Disrupts Eggshell Integrity and Retards Larval Development by Reducing Yolk Production in a *Caenorhabditis elegans* Model

**DOI:** 10.3390/nu12051334

**Published:** 2020-05-07

**Authors:** Hyemin Min, Esther Youn, Yhong-Hee Shim

**Affiliations:** Department of Bioscience and Biotechnology, Konkuk University, Seoul 05029, Korea; mintmin@konkuk.ac.kr (H.M.); dptmej@konkuk.ac.kr (E.Y.)

**Keywords:** caffeine, 1,3,7-trimethylxanthine, maternal effect, intergenerational effect, reproduction, yolk protein, vitellogenin, UNC-62, eggshell integrity, *Caenorhabditis elegans*

## Abstract

During pregnancy, most women are exposed to caffeine, which is a widely consumed psychoactive substance. However, the consequences of maternal caffeine intake on the child remain largely unknown. Here, we investigated the intergenerational effects of maternal caffeine intake on offspring in a *Caenorhabditis elegans* model. We treated a young mother (P0) with 10 mM of caffeine equivalent to 2–5 cans of commercial energy drinks and examined its reproduction and growth rate from P0 to F2 generation. The fertility decreased and embryonic lethality increased by defective oocytes and eggshell integrity in caffeine-ingested mothers, and F1 larval development severely retarded. These results were due to decreased production of vitellogenin protein (yolk) in caffeine-ingested mothers. Furthermore, effects of RNA interference of vitellogenin (*vit*) genes, *vit-1* to *vit-6*, in P0 mothers can mimic those by caffeine-ingested mothers. In addition, RNA interference (RNAi) depletion of *unc-62* (human Meis homeobox), a transcriptional activator for *vit* genes, also showed similar effects induced by caffeine intake. Taken together, maternal caffeine intake reduced yolk production mediated by the UNC-62 transcription factor, thereby disrupting oocyte and eggshell integrity and retarding larval development. Our study suggests the clinical significance of caffeine intake for prospective mothers.

## 1. Introduction

Caffeine is the most widely consumed bioactive molecule and its consumption has been increasing worldwide. A clinical issue about caffeine intake during pregnancy is emerging owing to the possible adverse impact of maternal nutritional status on child development [1,2]. However, the mechanism by which information is shared between the mother and child remains largely unknown.

*Caenorhabditis elegans* is an excellent animal model to study intergenerational effects of nutrient intake on the progeny because it is easy to examine embryonic and post-embryonic developmental processes in a large population of progeny at the organismal level. Furthermore, owing to its relatively small genome sequence and systematic phenotypic analyses, each process can be assessed at the molecular level as well [3,4]. *C. elegans* is also an excellent animal model to study reproduction because this hermaphrodite contains both egg and sperm. Thus, the entire reproductive progress from mitosis and meiosis of germ cells, and gametogenesis can be observed in one gonad arm. After the production of egg and sperm, the process of fertilization and even early embryogenesis are possibly observed simultaneously [5]. Several studies on caffeine intake in the *C. elegans* model have shown both beneficial and adverse effects on *C. elegans* development depending on the intake dose. At a high dose of caffeine (30 mM), stress responses were induced, larval development was inhibited, and even food-avoidance behavior was elicited when fed at the early larval stage of *C. elegans* [6,7,8]. However, at doses <10 mM of caffeine, the life span of *C. elegans* was extended [9,10]. Recent studies also suggest that caffeine has neuroprotective effects [11].

In this study, we examined reproduction in a caffeine-ingested mother, and consequently its embryonic and larval development in a *C. elegans* model. We found that 10 mM of caffeine intake caused defects in oocytes, increased embryonic lethality, and larval growth retardation. We further investigated the underlying molecular mechanisms and found that expression of *unc-62* gene (human Meis homeobox transcription factor) and its target genes, *vit* (vitellogenin) genes, was severely reduced. This resulted in defects in oocytes and intergenerational effects, including disrupted eggshell integrity and further retardation in larval development. Taken together, the results of this study suggest that caffeine intake by the mother can affect development of the progeny due to the reduction in yolk protein, which is a major source of nutrients in *C. elegans* oocytes and embryos.

## 2. Materials and Methods

### 2.1. Caenorhabditis Elegans Strains and Caffeine Treatment

*Caenorhabditis elegans* strains were maintained at either 15 or 20 °C on nematode growth medium (NGM) agar plates seeded with *Escherichia coli* strain OP50, as described previously [12]. The following strains were used in the present study: N2 (*C. elegans* wild isolate, Bristol variety), RB1982: *vit-1(ok2616) X,* RB2365: *vit-2(ok3211) X,* RB2382: *vit-5(ok3239) X,* DH1033: *bIs1 (vit-2::GFP+rol-6(su10060)) X,* and BC12843: *dpy-5(e907) I; sIs11286(rCesK07H8.6(vit-6)::GFP+pCeh361).* To examine the effects of caffeine intake, caffeine (Sigma-Aldrich, St. Louis, MO, USA) was added to NGM before autoclaving to obtain final concentrations of 5, 10, and 30 mM caffeine. Synchronized L4-stage animals were exposed to caffeine for 24 h at 20 °C and then the adult-stage mothers and their progenies were examined.

### 2.2. Analysis of the Number of Progenies, Embryonic Lethality, and Percent Larval Development

L4-stage wild-type N2 hermaphrodites were individually cloned onto either caffeine-containing (5–30 mM) or caffeine-free (0 mM) NGM agar plates and grown at 20 °C. They were transferred to new plates in 24 h intervals for 4 days to allow embryo production. Laid embryos were considered dead if they did not hatch after 48 h at 20 °C. The number of progenies was calculated as the total number of non-hatched and hatched embryos produced by a single mother. Embryonic lethality was calculated as the percentage of non-hatched embryos of the total number of embryos produced for 4 days. Percent larval development was calculated as the percentage of larvae of the total number of hatched embryos that reached each developmental stage, as previously described [6]. We distinguished the developmental larval stages as follows: L1, the smallest larvae <0.3 mm; L2, larvae larger than L1 (body length, 0.3–0.4 mm) but with no characteristics of L3; L3, larvae with a white spot in the vulva region (body length, 0.4–0.6 mm); L4, larvae with a characteristic half-moon-like shape in the vulva region (body length, 0.6–0.8 mm); adults, animals with an opened vulva with eggs in the uterus.

### 2.3. Western Blot Analysis

Western blot analysis was performed using whole animal protein extract obtained from ca. 200 gravid adult hermaphrodites of each condition per gel well. Antibodies bound to a nitrocellulose membrane (PROTRAN BA83, Whatman, Sigma-Aldrich, St. Louis, MO, USA) were visualized with Chemiluminescence (ECL) Western blotting detection kit (Amersham, GE Healthcare Life Sciences, Pittsburgh, PA, USA), and the respective band intensities were measured with LAS-3000 image analyzer using Multi Gauge software (v.3.0, Fuji Film, Tokyo, Japan). The following primary and secondary antibodies were used: rabbit anti-GFP (1:1000, Novus, St. Charles, MO, USA), mouse anti-α-tubulin (1:1000; Sigma-Aldrich, St. Louis, MO, USA), HRP-conjugated goat anti-rabbit IgG (1:1000; Santa Cruz Biotechnology, Dallas, TX, USA), and HRP-conjugated donkey anti-mouse IgG (1:1000; Jackson ImmunoResearch, PA, USA).

### 2.4. Analysis of Oocyte and Eggshell Integrity

To investigate oocyte and eggshell integrity after caffeine intake, membrane permeability was assessed using FM4-64 dye (Sigma-Aldrich, St. Louis, MO, USA), as previously described [13]. In brief, caffeine-ingested mothers were dissected in 150 mM KCl with 30 µM of FM4-64 dye to observe oocytes and embryos. The proportion of either embryos or oocytes infiltrated by FM4-64 was measured using a Zeiss microscope at 40× magnification. For each case, three independent experiments were performed.

### 2.5. RNA Interference (RNAi) Assays

RNAi experiments were performed using the soaking method, as previously described [14]. dsRNAs of *vit-1, vit-2, vit-3, vit-4, vit-5, vit-6,* and *unc-62* genes were synthesized in vitro using the respective cDNA template. The cDNA templates flanked by T7 promoter sequences were generated by PCR using T7 primer, 5′-GTAATACGACTCACTATAGGGC-3′ and CMo422 primer, 5′-GCGTAATACGACTCACTATAGGGAACAAAAGCTGGAGCT-3′. Soaking buffer without dsRNA was used as the negative mock RNAi control. L4-stage animals were soaked in dsRNA solution for 24 h, then transferred onto caffeine-containing NGM agar plates to grow for 24 h until the animals reached the adult stage. The adult-stage animals were evaluated by membrane integrity assay.

### 2.6. DNA Staining in Oocytes

To observe whether oocytes of caffeine-ingested mothers have six pairs of homologous chromosomes (bivalents), DNA staining was performed, as previously described [14]. Animals were dissected to extrude gonads in 10 µL of M9 buffer containing 100 µg/mL tetramisole on a poly-L-lysine-coated slide, covered with a coverslip, freeze-cracked with liquid nitrogen, and fixed with cold methanol and cold acetone. The specimens were then stained with 1 µM TO-PRO-3 (Molecular Probes, Eugene, OR, USA) for 1 h at 20 °C to stain DNA and then observed under a confocal microscope (Olympus, FV1000 Spectral, Tokyo, Japan).

### 2.7. Real Time RT-PCR (qRT-PCR)

Adult hermaphrodites of wild-type that were treated or not treated with caffeine (10 mM) were collected in TRIzol (Invitrogen, Waltham, MA, USA), and total RNA was extracted using a phase lock gel (MaXtract High Density, Qiagen, Germantown, MD, USA). cDNA was synthesized using oligo-dT primer and M-MLV reverse transcriptase (Invitrogen, Waltham, MA, USA). qRT-PCR assays were performed using SYBR Green PCR Master Mix (Applied Biosystems, Waltham, MA, USA). The final PCR volume was 10 µL. *act-1* mRNA was used as an endogenous control for data normalization. The primers used for the measurement of expression of the *unc-62* gene were as follows: forward, 5′-TAAGACATACCCAAGAGAATGCTG-3′ and reverse, 5′-TTTGCCTTTCAGACAGACCA-3′.

### 2.8. Statistical Analysis

All experiments were repeated more than three times for statistical evaluation of the data. Two-tailed Student’s *t*-test was used to calculate *p-*values; *p* < 0.05 was considered significant. The data are expressed as the mean ± standard deviation (SD).

## 3. Results

### 3.1. Maternal Caffeine Intake Causes a Reduction in Fertility and Retardation in the Developmental Growth of Progeny in C. elegans

**Figure 1:**
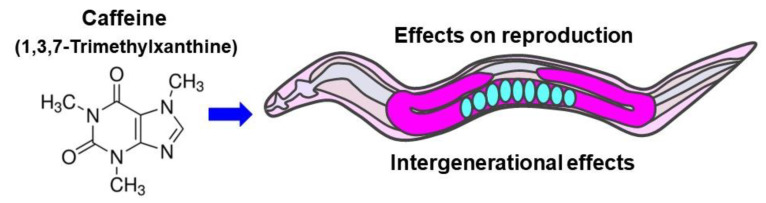
*Caenorhabditis elegans* P0 mothers were fed single compound caffeine (1,3,7-trimethylxanthine).

**Figure 2:**
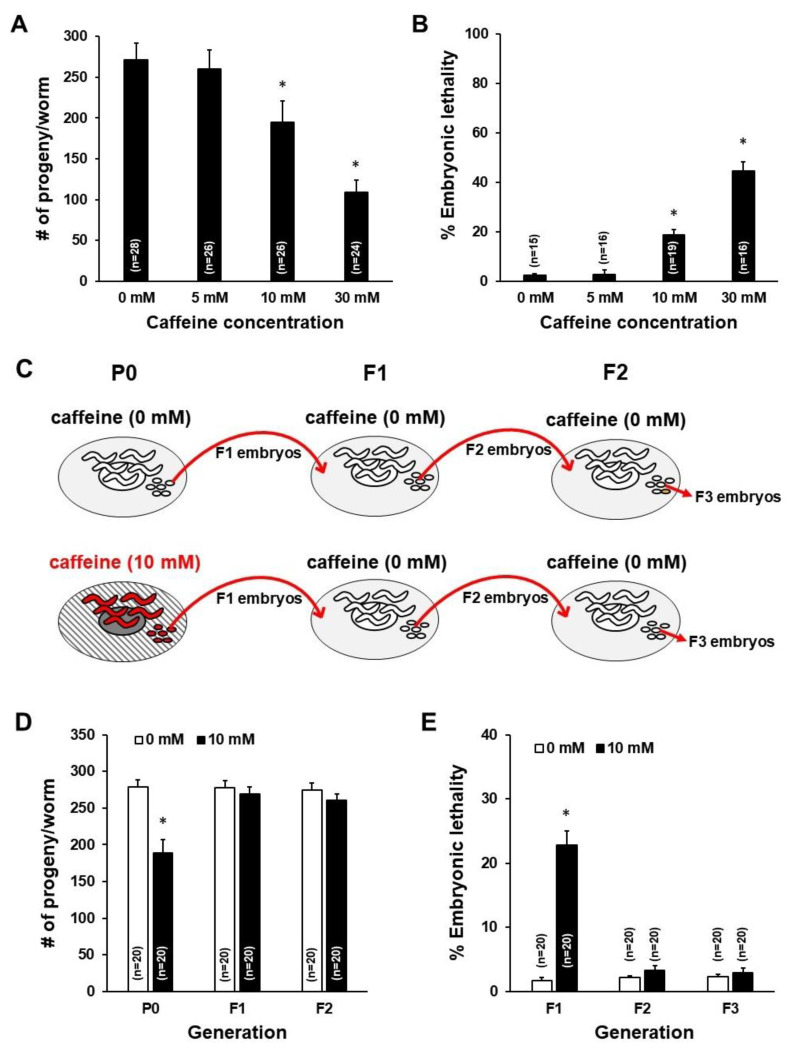
Caffeine intake caused a reduction in fertility of P0 mothers and an increase in F1 embryonic lethality in *Caenorhabditis elegans*. (**A**) Total number of progenies by caffeine-ingested mothers (5, 10, and 30 mM) compared to mothers with a caffeine-free diet (0 mM). * *p* < 0.05. (**B**) The percentage of embryonic lethality among the total number of progenies produced by caffeine-ingested P0 mothers that were fed 0, 5, 10, and 30 mM of caffeine at the L4 stage for 24 h. * *p* < 0.05. (**C**) A scheme of assays for intergenerational effects of caffeine intake by P0 mothers. The P0 mothers were fed 10 mM of caffeine, and reproduction and growth were measured from P0 to F2 generation for analysis of intergenerational effects of caffeine intake by P0 mothers. (**D**) The total number of progenies produced by caffeine-ingested P0 mothers and their offspring (black bars) compared to caffeine-free diet P0 mothers and their offspring (white bars). * *p* < 0.05. (**E**) The percentage of embryonic lethality among the total number of progenies produced by caffeine-ingested P0 mothers and their offspring (black bars), and caffeine-free diet P0 mothers and their offspring (white bars). Error bars represent SD. * *p* < 0.05.

To investigate whether caffeine intake by the mother has intergenerational effects on offspring, we fed caffeine only to P0 mothers, as shown in Figure 1, and measured the fertility and the developmental growth of offspring. It has been previously reported that the effects of caffeine treatment are dose dependent [6]. Therefore, we first examined the effect of doses of caffeine intake on reproduction by feeding 0, 5, 10, and 30 mM of caffeine to hermaphrodites of wild-type L4-stage animals for 24 h. The number of progenies was significantly decreased and embryonic lethality was increased when mothers were fed 10 or 30 mM of caffeine (Figure 2A,B). These results indicate that >10 mM of caffeine intake seriously reduced fertility. In this study, the effects of 10 mM of caffeine intake were examined in the subsequent experiments because some mothers fed 30 mM of caffeine became sick.

To determine whether caffeine intake by the mother has intergenerational effects, fertility including the number of progenies and embryonic lethality was assessed from P0 to F2 generation in *C. elegans* (Figure 2C). The number of progenies decreased in P0 mothers fed 10 mM caffeine, but not in F1 and F2 generation mothers. In addition, embryonic lethality increased in F1 embryos produced by the caffeine-ingested P0 mother, but not in F2 and F3 embryos (Figure 2D,E). These results suggest that caffeine intake reduced fertility in P0 mothers, but not in F1 or F2 mothers.

**Figure 3:**
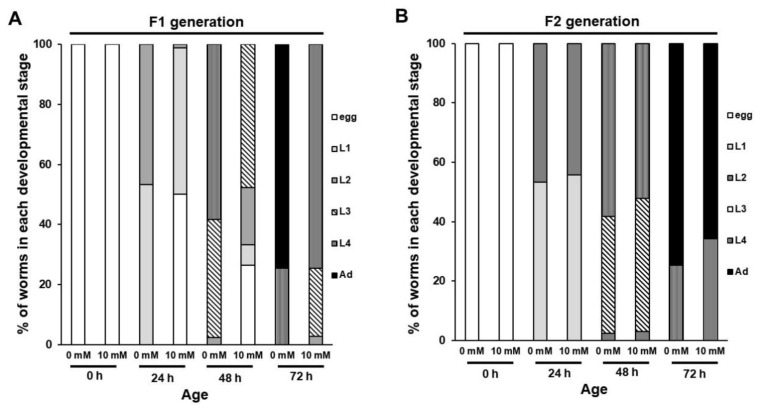
Caffeine intake by P0 mothers affected development in the subsequent generation of *Caenorhabditis elegans*. (**A**,**B**) Synchronized L4-stage animals (*n* = 30) of *C. elegans* wild-type were fed either 0 or 10 mM of caffeine for 24 h, and embryos were transferred to respective OP50-seeded nematode growth medium plates and further cultured at 20 °C. The developmental stage of each individual in the F1 and F2 generations was determined based on its size and stage-specific morphological characteristics (see Section 2 for details) during development by either caffeine-ingested (10 mM) or caffeine-free diet P0 mother (0 mM).

Next, we tested the possibility that caffeine intake could affect growth of hatched offspring produced by caffeine-ingested mothers. We evaluated the developmental growth rate in F1 and F2 generations of caffeine-ingested P0 mothers. Interestingly, caffeine-ingested P0 mothers showed a significantly retarded developmental growth rate in the F1 generation (Figure 3A). However, in the F2 generation, no growth retardation was observed (Figure 3B). These results suggest that caffeine intake by the mother delays growth of the subsequent F1 generation (ca. 70% adult without caffeine intake, but 0% adult with caffeine at 72 h growth), but not of the F2 generation (ca. 70% adult in both 0 mM and 10 mM caffeine intake at 72 h growth).

### 3.2. Maternal Caffeine Intake Reduces Yolk Production in C. elegans

**Figure 4:**
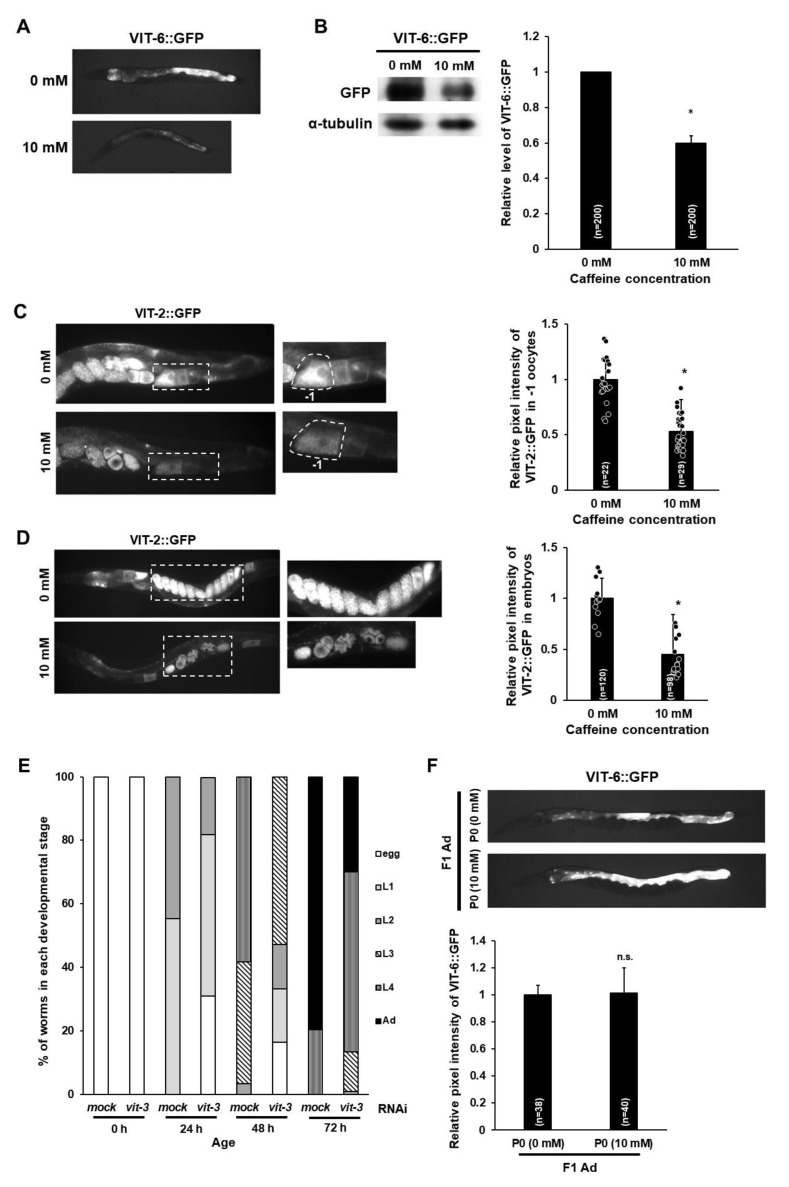
Maternal caffeine intake reduces vitellogenin (yolk proteins) in *Caenorhabditis elegans*. (**A**) VIT-6::GFP transgenic animals synchronized at the L4-stage were exposed to caffeine for 24 h at 20 °C. VIT-6::GFP expresses in the intestine. The reduced level of VIT-6::GFP in the intestine was observed in caffeine-ingested mothers. (**B**) Western blot analysis of VIT-6::GFP protein levels in caffeine-free diet mother (0 mM) and caffeine-ingested mother (10 mM). Respective GFP band intensities were normalized against those of α-tubulin in the same lane. Then the normalized GFP band intensity was converted to a relative value compared to the normalized GFP band intensity of 0 mM, as shown in the right graph with mean ± SD values. These GFP band intensity values were obtained from three independent Western blot analyses. Statistical significance was calculated using Student’s *t*-test. * *p* < 0.05. (**C**,**D**) VIT-2::GFP transgenic animals synchronized at the L4-stage were exposed to caffeine for 24 h at 20 °C. The caffeine-ingested mother showed a reduced level of VIT-2::GFP intensity in oocytes and embryos, as shown in the right graph with mean ± SD values. (**E**) Synchronized L4-stage animals (*n* = 30) of wild-type were treated with RNA interference (RNAi) of *vit-3* gene for 24 h and recovered to OP50-seeded nematode growth medium (NGM) plates for 24 h. The embryos produced by P0 mothers were transferred to respective NGM plates and further cultured at 20 °C. The developmental stage of each individual in the progenies was determined based on its size and stage-specific morphological characteristics (see Section 2 for details) during development by either the *vit-3* RNAi-treated P0 mother (10 mM) or non-treated (mock) P0 mother (0 mM). (**F**) VIT-6::GFP transgenic animals synchronized at the L4-stage were exposed to caffeine for 24 h at 20 °C. Then their F1 generation grew under the caffeine-free diet (0 mM) condition, and VIT-6::GFP transgenic F1 animals were observed at the adult stage. Statistical significance was calculated using Student’s *t*-test. n.s., *p* > 0.05.

Vitellogenin (yolk proteins) plays an important role in the normal development of the animal’s offspring by supplying nutrients [15,16,17]. To understand the molecular mechanism underlying the retarded growth rate of offspring produced by caffeine-ingested mothers, we investigated the possibility of association with the expression of vitellogenin. We measured the expression level of vitellogenin gene 6 (*vit-6*) and vitellogenin gene 2 (*vit-2*) after caffeine intake using transgenic animals with a transgene *vit-6::gfp* or *vit-2::gfp*. Adult-stage transgenic animals expressing either VIT-6::GFP or VIT-2::GFP were examined following exposure of caffeine and expression levels of both VIT-6::GFP and VIT-2::GFP were found to be reduced (Figure 4A–D). We confirmed a significantly reduced level of VIT-6::GFP compared to the control by Western blot analysis (Figure 4B). VIT-2 and VIT-6 are exclusively expressed in the intestine at the adult stage and VIT-2 is transported into oocytes and eventually to the embryonic cells, whereas VIT-6 remains in the intestine [18]. We thus observed oocytes and embryos for VIT-2::GFP expression and found a significantly decreased level of VIT-2::GFP both in oocytes and embryos after caffeine intake (Figure 4C,D). These results demonstrate that the expression of vitellogenin genes is suppressed by caffeine intake.

We further assessed a possible association between the reduction in vitellogenin and developmental growth of offspring by examining the developmental stages of F1 worms produced by P0 mothers treated with RNA interference (RNAi) of the vitellogenin gene *vit-3*. We observed a retarded developmental growth rate in *vit-3* RNAi depleted worms (Figure 4E), indicating that the presence of vitellogenin is required for the normal growth of offspring. Taken together, these results suggest that caffeine intake by the mother reduces the production of vitellogenin, which causes retarded growth of offspring. We further examined whether the reduced level of vitellogenin observed in caffeine-ingested P0 mothers sustains in the adult-stage F1 offspring. Interestingly, F1 generation adults expressed VIT-6::GFP similarly to that in the caffeine-free diet control group (Figure 4F), suggesting that the effect of reduced levels of vitellogenin on the development of offspring is limited to F1 generation. These findings suggest that the decreased level of vitellogenin in the caffeine-ingested mother caused retarded larval growth of the F1 offspring, but the expression of vitellogenin in the adult stage of the F1 generation was not altered and no further effects were observed in F2 generation (Figure 3B, Figure 4F).

### 3.3. Maternal Caffeine Intake Disrupts Eggshell Integrity in C. elegans

**Figure 5:**
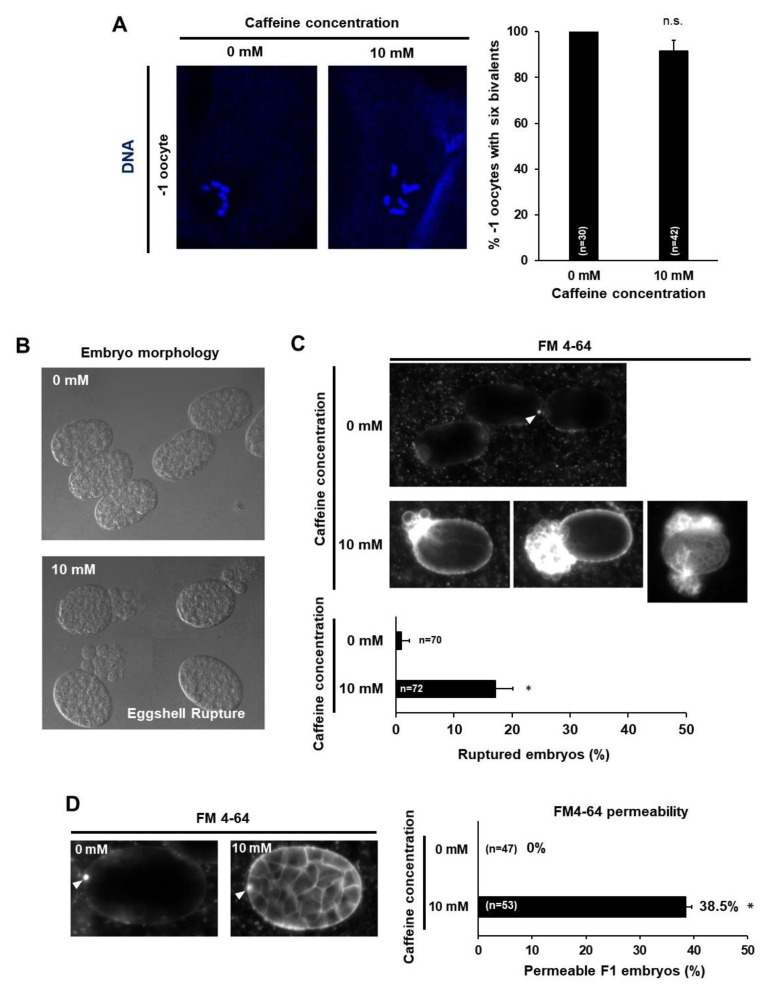
Maternal caffeine intake disrupted eggshell integrity in *Caenorhabditis elegans*. (**A**) Oocytes at the –1 position in a gonad arm of wild-type adult hermaphrodites grown without or with 10 mM caffeine. DNA was stained with TO-PRO-3 and then six bivalents were examined. The percent of intact six bivalents is shown in a bar graph on the right. (**B**–**D**) DIC images (**B**) and lipophilic dye FM4-64 staining images (**C**,**D**) of embryos from the dissected caffeine-free diet (0 mM) and caffeine-ingested mothers (10 mM). The caffeine-free diet (0 mM) mother produced intact and ovoid embryos and only polar bodies (white arrowheads) were stained, but the caffeine-ingested mother produced ruptured embryos (**C**) and their eggshells were permeable to FM4-64, and the cell membranes of the embryos were stained (**D**). Statistical significance was calculated using Student’s *t*-test. * *p* < 0.05.

Caffeine-ingested mothers showed an increased level of embryonic lethality (Figure 2B). Chromosomal alterations in oocytes have been reported as the primary cause of embryonic lethality in *C. elegans* [19]. Therefore, we examined DNA morphology in 𢈒1 position oocytes of caffeine-ingested mothers under a fluorescent microscope after DNA staining with TO-PRO-3 fluorescent dye. The majority of mature oocytes in caffeine-ingested mothers contained six pairs of aligned and condensed chromosomes in their nuclei, which are characteristic of the diakinesis stage in meiotic prophase I in normal −1 position oocytes (Figure 5A). As little as 8.4% of oocytes showed a chromosomal abnormality, which was not statistically significant (*p* = 0.068). This result indicates that caffeine intake by mothers did not affect chromosomal integrity in oocytes.

The eggshell, which provides a protective structure with the extracellular matrix, is an important factor in many early developmental events [13,20] and recent studies have suggested the pivotal role of the eggshell during embryonic development and survival [21,22]. Therefore, we next examined eggshell integrity in F1 embryos produced by caffeine-ingested mothers. We isolated embryos from dissected caffeine-ingested and caffeine-free diet mothers and examined their integrity. Approximately 17% of embryos isolated from caffeine-ingested mothers were ruptured (Figure 5B,C); the remaining embryos were examined with lipophilic dye FM4-64 staining for visualizing embryonic morphology [13,23]. Surprisingly, approximately 38% of the embryos out of the 83% non-ruptured embryos from caffeine-ingested mothers were permeable to FM4-64 and their embryonic membrane was stained, whereas embryos produced by caffeine-free diet mothers were not permeable to FM4-64 and their embryonic membrane was not stained at all (Figure 5D). Lipophilic FM4-64 dye binds to the embryonic membrane and stains it if the eggshell is disrupted. Taken together, maternal caffeine intake disrupted eggshell integrity, causing the eggshell to rupture and become permeable.

### 3.4. Reduced Vitellogenin Production in Caffeine-Ingested Mothers Causes Defective Oocyte and Eggshell Integrity in C. elegans

**Figure 6:**
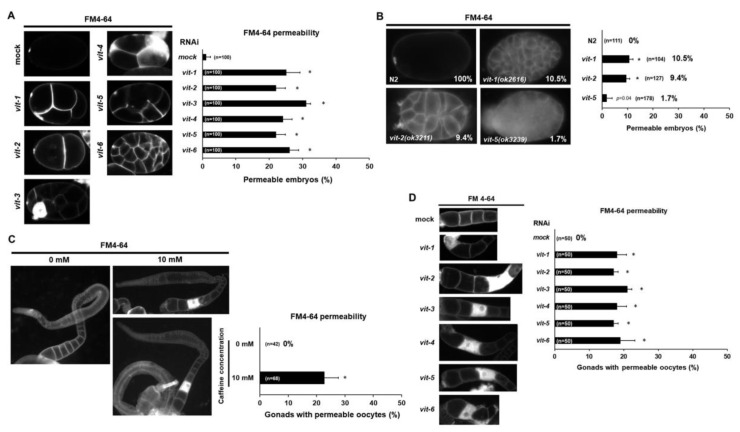
Vitellogenin is required for eggshell and oocyte integrity in *Caenorhabditis elegans*. (**A**) Eggshell permeability was examined by lipophilic dye FM4-64 staining in the embryos produced by mock RNAi- or *vit-1 to vit-6* RNAi-treated mothers. Statistical significance was calculated using Student’s *t*-test. * *p* < 0.05 against mock RNAi-treated animals. (**B**) Eggshell permeability was examined by FM4-64 staining in the embryos produced by *vit-1(ok2616), vit-2(ok3211),* and *vit-5(ok3239*) mutants. Statistical significance was calculated using Student’s *t*-test. * *p* < 0.05 against N2 control. (**C**) Oocyte permeability examined with lipophilic dye FM4-64 staining in the caffeine-ingested mother (10 mM) and caffeine-free diet mother (0 mM). Gonads were extruded by dissecting adult animals in 150 mM of KCl. Statistical significance was calculated using Student’s *t*-test. * *p* < 0.05. (**D**) Oocyte permeability was examined by FM4-64 staining after treatment with RNAi of the vitellogenin genes from *vit-1 to vit-6*. Statistical significance was calculated using Student’s *t*-test. * *p* < 0.05 against mock RNAi-treated animals.

We found that caffeine intake reduced vitellogenin production (Figure 4A–D) and disrupted eggshell integrity (Figure 5B–D). On the basis of these findings, we investigated the relationship between vitellogenin and eggshell integrity to determine whether disrupted eggshell integrity could be due to the reduction in vitellogenin production. The vitellogenin family is comprised of six genes: *vit-1* to *vit-6* [24]. We performed the respective RNAi of the six *vit* genes to knock down vitellogenin production and observed eggshell permeability of embryos produced by RNAi-treated mothers (Figure 6A). Approximately 20% of embryos were permeable to FM4-64 by depletion of *vit* genes, whereas a majority of mock RNAi-treated embryos were not permeable to FM4-64 (Figure 6A). The eggshell permeability defects in the *vit* RNAi experiments were likely due to simultaneous knockdown of more than one *vit* gene, due to the RNAi target sequence similarity to more than one *vit* gene. Off target effects likely occur because *vit-1* is 82% identical to *vit-2*, *vit-3* and *vit-4* are 99% identical, *vit-5* is 96% identical to *vit-3*, and *vit-6* is 50% identical to *vit-2* [17]. Indeed, a single *vit* mutant only showed subtle phenotype, suggesting the functional redundancy among *vit* genes. We observed that approximately 10% of embryos in *vit-1(ok2616)* and *vit-2(ok3211)* mutants, and approximately 1.7% of embryos in *vit-5(ok3239)* mutant were permeable to FM4-64 dye (Figure 6B). Here we presented results from *vit* RNAi and mutant analyses (Figure 6), which suggest that the reduction in yolk proteins disrupts eggshell integrity.

Next, we examined whether disrupted eggshell integrity is associated with oocytes in caffeine-ingested mothers. We hypothesized that disruption in eggshell integrity is possibly due to defective oocytes in caffeine-ingested mothers and thus we examined permeability of oocytes using lipophilic dye FM4-64. Interestingly, caffeine-ingested mothers exhibited approximately 20% of gonads with permeable oocytes, unlike oocytes produced by caffeine-free diet mothers (Figure 6C). Furthermore, we also performed RNAi of respective vitellogenin genes to test whether the reduction in vitellogenin can also allow oocytes to be permeable. The respective vitellogenin gene (*vit-1*, *-2*, *-3*, *-4*, *-5*, and *-6*) RNAi-treated mothers produced approximately 18–22% of permeable oocytes in gonads (Figure 6D). These findings indicate that maternal caffeine intake caused defective oocytes and disrupted eggshell integrity through the reduction in vitellogenin production.

### 3.5. Maternal Caffeine Intake Reduces unc-62 Expression and the Reduced Level of unc-62 Causes Defective Oocyte and Eggshell Integrity in C. elegans

**Figure 7:**
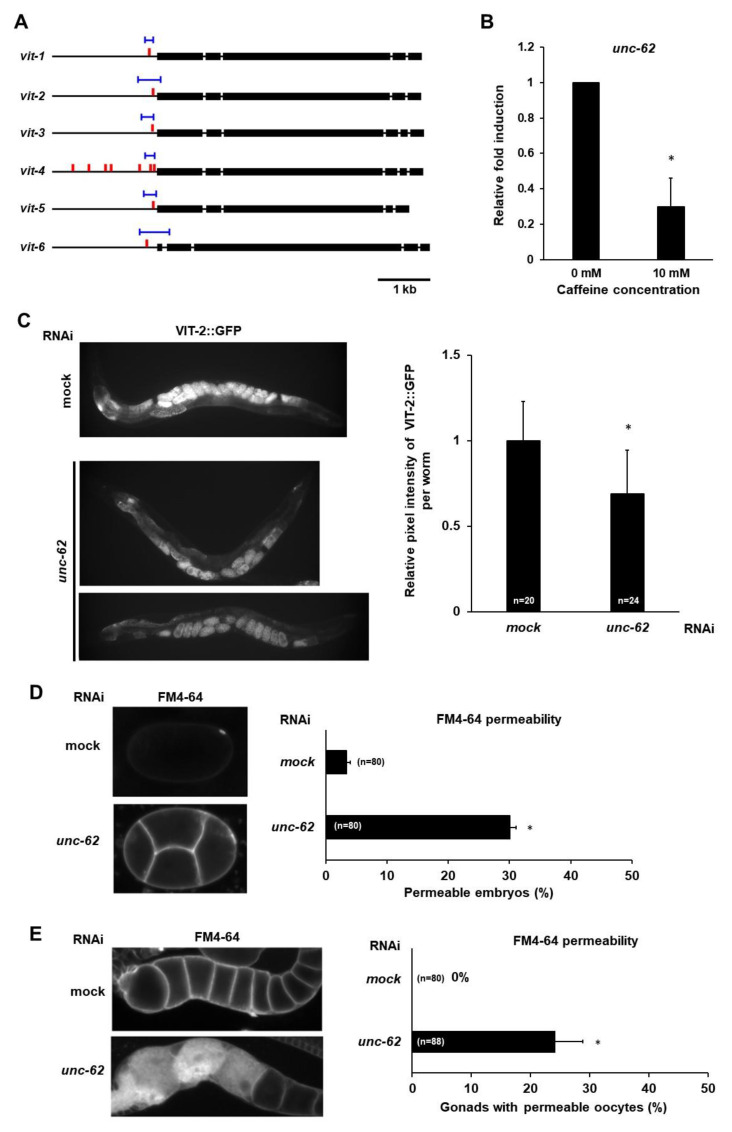
Caffeine intake reduces *unc-62* expression and the reduced level of *unc-62* exhibits defects in embryo and oocyte integrity in *Caenorhabditis elegans*. (**A**) UNC-62 binding sites in the genomic structures of vitellogenin genes. The thin black lines indicate the promoter and introns of each of six vitellogenin loci, and the thick black lines indicate exons of six vitellogenin genes. The blue lines indicate UNC-62 binding sites, and the red bars indicate VPE-1 (ATTGACA) vitellogenin regulatory motif previously described [25,27]. (**B**) Fold induction of mRNA level of *unc-62* in caffeine-ingested mothers (10 mM) than caffeine-free diet mothers (0 mM). The mRNA level of *unc-62* was determined by three independent qRT-PCR using the mRNA level of *act-1* in each sample as an internal control for normalization. T-bars represent SD. Approximately 150 adult animal individuals were used to prepare total RNA for respective conditions. Statistical significance was calculated using Student’s *t*-test. * *p* < 0.05. (**C**) VIT-2::GFP transgenic animals synchronized at the L4-stage were treated with *unc-62* RNAi. The unc-62 RNAi-treated mothers showed the reduced level of VIT-2::GFP intensity both in oocytes and embryos, as shown in the right graph with mean ± SD values. (**D**) Eggshell permeability examined by lipophilic dye FM4-64 staining of the embryos produced by *unc-62* RNAi-treated mothers. Statistical significance was calculated using Student’s *t*-test. * *p* < 0.05 against mock RNAi-treated animals. (**E**) Oocyte permeability examined by lipophilic dye FM4-64 staining in the dissected gonad from the *unc-62* RNAi-treated mothers. Statistical significance was calculated using Student’s *t*-test. * *p* < 0.05 against mock RNAi-treated mothers.

It has been reported that VPE1 (TGTCAAT) and VPE2 (CTGATAA), the *cis*-elements in the *vit* promoter, are important for vitellogenin (*vit*) gene expression (Figure 7A), [25,26]. VPE1 is bound by the UNC-62 (human Meis homeobox) transcription factor, which is highly expressed in the intestines of adult *C. elegans*, where *vit* genes are specifically expressed; and expression of *vit* genes is suppressed in *unc-62* RNAi-treated animals [27]. Therefore, we investigated whether *unc-62* expression is affected by caffeine intake by measuring the mRNA level of *unc-62* gene in caffeine-ingested and caffeine-free diet mothers using qRT-PCR (Figure 7B). We found that the mRNA level of *unc-62* gene significantly decreased in caffeine-ingested mothers (Figure 7B) and the reduced *unc-62* level by RNAi led to a decreased level of VIT-2::GFP (Figure 7C). These results suggest that the reduced vitellogenin production by caffeine intake is caused by the decreased level of transactivator *unc-62*. 

**Figure 8:**
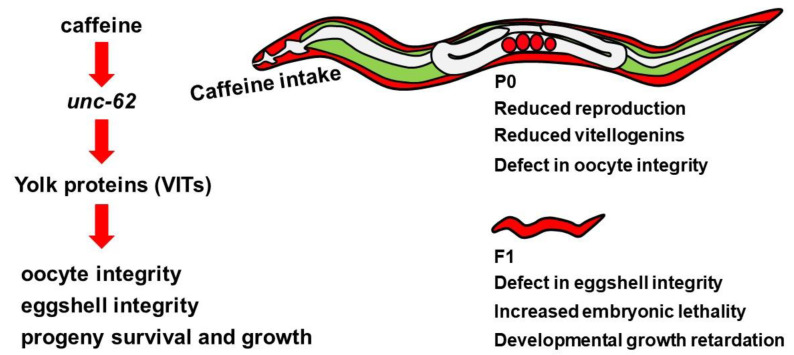
Model of the intergenerational effects of maternal caffeine intake in *Caenorhabditis elegans*. Caffeine intake decreased the production of yolk proteins by reducing *unc-62* expression. The decreased levels of yolk proteins disrupted oocyte and eggshell integrity and induced embryonic lethality and growth retardation of the next generation.

Next, we addressed whether the reduced level of *unc-62* can cause defects in eggshell and oocyte integrity. We performed *unc-62* RNAi in mothers and observed eggshell and oocyte integrity by FM4-64 dye staining. The *unc-62* RNAi-treated embryos and oocytes also became permeable to FM4-64 dye (Figure 7C,D), indicating that the reduced level of *unc-62* induced by caffeine intake results in defective eggshell and oocyte integrity possibly through the reduction in vitellogenin production. Taken together, we propose that maternal caffeine intake affects the survival and growth of offspring through the reduction in yolk protein production, which is mediated by the repression of *unc-62* gene expression (Figure 8).

## 4. Discussion

The physiological effects of caffeine intake in *C. elegans* are highly dose dependent [6]. Previous reports about the effects of caffeine using a *C. elegans* model indicate that the intake of a high dose of caffeine (>10 mM) showed adverse effects, such as developmental arrest, activation of stress-response pathways, and stimulation of food-avoidance behavior [6,7,8], whereas animals treated with a low dose of caffeine (<10 mM) generally showed beneficial effects such as lifespan extension, antioxidant effects, and protection of neurodegeneration [10,28,29]. However, the mechanism by which maternal caffeine intake affects not only the mother but also the offspring remains largely unknown. In this study, we examined the intergenerational effects of caffeine intake (10 mM) by mothers on reproduction and offspring development in a *C. elegans* model. We found that 10 mM of caffeine intake by the mother caused reduced fertility with defective oocytes and eggshell integrity, and an increased level of embryonic lethality and retardation in larval development (Figure 2A,B; Figure 3A).

There are six *vit* (vitellogenin) family genes and their expressions are stage-, sex-, and tissue-specific in *C. elegans*, which are exclusively and highly expressed in the adult hermaphrodite intestine [24]. Therefore, *vit* gene expression should be regulated by specific transcription factors. There are two major binding sites known as VPE1 and VPE2 in all six *vit* genes in *C. elegans* [25]. UNC-62, a VPE1-binding transcription factor, is highly expressed in the intestinal nuclei during the adult stage of *C. elegans* observed by UNC-62::GFP, and it activates *vit* gene expression [27]. *unc-62* is an ortholog of human MEIS1 (Meis homeobox 1) and MEIS2 transcription factor that functions in normal human development [30]. In this study, we found that *unc-62* expression was repressed by caffeine intake. How does caffeine intake regulate *unc-62* expression? It has been reported that caffeine intake influences metabolic rate and lipid oxidation [31,32,33]. In addition, it was suggested that nutrients can control transcription factors [34]. Since *unc-62* appears to be an upstream gene to control the developmental processes as a homeobox transactivator, this gene is possibly regulated by the nutritional state during the development of *C. elegans.* Considering that caffeine intake modulates the metabolic pathways and changes nutritional state in an animal, the developmental regulators are the possible targets responding to the alterations in metabolism. To investigate this possibility, the changes in the metabolic pathways in caffeine-ingested mother and the relation between these pathways and *unc-62* repression needs to be determined. Vitellogenin (YP170) is transported from the intestine, where it is produced, to the oocytes by receptor-mediated endocytosis; it becomes enriched in the yolk and provides nutrients to the next generation in *C. elegans* [35]. Therefore, vitellogenin is considered as an intergenerational molecule that is transferred from the mother to the offspring. Suppression of *vit* gene expression after caffeine intake suggests that food signals are possibly involved in vitellogenesis. *C. elegans vit* genes are homologous to those in vertebrates [36]. In vertebrates, vitellogenin is produced in the female liver responding to estrogen signals and transported to the oocytes in the ovary through the blood. Furthermore, environmental estrogenic chemicals can induce vitellogenin production. Therefore, vitellogenin production is sensitive to the environmental status; thus, it can be used as a biomarker of environmental stress during reproduction [37]. The novel finding that vitellogenin is required for eggshell integrity and lack of vitellogenin causes embryonic lethality in approximately 20% of the embryos observed in this study suggests that the presence of vitellogenin is important for embryonic development of the next generation. Vitellogenin is mainly associated with lipid droplets in which phosphatidylcholine (PC, 23%) and phosphoethanolamine (PE, 28.2%) are over half of the total lipid content [17]. PC and PE are a major class of phospholipids that are the main constituents of the cell membrane [38]. Therefore, the reduction of vitellogenin along with PC and PE in oocytes may cause defects in the vitelline layer of oocytes and permeability. However, we propose that vitellogenin can partially contribute to the survival of embryos and other factors can compensate for the requirement of vitellogenin during embryogenesis. This is supported by the findings that 23% of live embryos were retained even with complete loss of vitellogenin in oocytes in *rme-2* mutants [35]; and that vitellogenin production was remarkably decreased under dietary restriction in *C. elegans*, causing increased embryonic lethality that was suppressed by methionine supplement [39]. In addition, larval growth retardation in the F1 generation of caffeine-ingested mothers in the present study reveals that vitellogenin is indeed an intergenerational protein. A large amount of vitellogenin from the mother was observed in the early F1 larval stage of *C. elegans*, suggesting that maternal vitellogenin remains in the larval stage and provides nutrients for larval development in the F1 progeny [40]. However, in the present study, we found that the decreased level of vitellogenin in the mother affected the F1 generation, but not further generations, suggesting that it did not affect vitellogenin production in the adult-stage intestine and germ cells in the F1 generation.

The eggshell plays an important role in protecting the embryo by maintaining proper osmotic conditions and preventing the entry of potentially harmful molecules from the environment. In addition, the permeability barrier of the embryo also allows it to maintain the substances required for embryogenesis [41,42,43]. Previous studies have shown that fatty acid synthesis and modifications of enzymes are required for the formation of the permeability barrier in the embryo [23], and lipid metabolism is strongly associated with eggshell integrity [43]. It has been reported that in zebrafish, caffeine intake has a role in the suppression of fatty liver by downregulation of genes associated with lipogenesis, and enhancement of lipid oxidation and autophagy activity [44]. A relationship between caffeine intake and fat metabolism has also been observed in a rat model system, which has shown that caffeine intake reduces body fat by lipolysis and has an anti-obesity effect [45]. These findings suggest that caffeine intake affects lipid metabolism, which in turn possibly controls eggshell formation. Therefore, in addition to vitellogenin production, lipid metabolism appears to be involved in eggshell and oocyte integrity after caffeine intake. Further analysis of the direct relationship between caffeine intake and lipid metabolism in eggshell integrity remains to be determined. Dietary habit is one of the external stimulations to induce internal physiological effects. Caffeine intake can be an effective external stimulator. It has previously been described that caffeine intake causes global deacetylation of proteins and mimics caloric restriction through autophagy induction [46]. It will be worthwhile to examine whether the mode of action in oocyte and eggshell integrity after caffeine intake is related to autophagy.

In summary, our results suggest that caffeine intake by the mother affects reproduction in a dose-dependent manner. We demonstrated that maternal caffeine intake reduces yolk production by regulating *unc-62* expression. The reduced vitellogenin production by caffeine intake, in turn, decreases embryonic survival by disrupting eggshell integrity, and inhibits larval development. As reported, vitellogenin expression is highly specific at the adult stage of *C. elegans* females. Therefore, further investigation of the consequences of caffeine intake in males to understand paternal effects is required.

## 5. Conclusion

This study provides several evidences showing the intergenerational effects of maternal caffeine intake. These effects were attributed to the suppression of yolk protein production mediated by a transcriptional activator, *unc-*62 (human Meis homeobox). These findings support that the mother’s diet during pregnancy is critical for the survival and growth of progeny.
